# Le synovialosarcome de la sphère oto-rhino-laryngée: une localisation rare: à propos de deux cas

**DOI:** 10.11604/pamj.2015.20.232.6225

**Published:** 2015-03-12

**Authors:** Fadila Kouhen, Mohammed Afif, Naoual Benhmidou, Fadoua Rais, Mustapha El kabous, Mouna Khmou, Nadia Cherradi, Sanaa Majjaoui, Hanan Elkacemi, Tayeb Kebdani, Noureddine Benjaafar

**Affiliations:** 1 Service de Radiothérapie, Institut National d'Oncologie, Université Mohammed V, Rabat, Maroc; 2 Service d'Oncologie Médicale, Institut National d'Oncologie, Université Mohammed V, Rabat, Maroc; 3 Service d'Anatomopathologie, Hôpital des Spécialités, Université Mohammed V, Rabat, Maroc

**Keywords:** Synovialosarcome, ORL, chirurgie, radiothérapie, Synovialosarcoma, ENT, surgery, radiotherapy

## Abstract

La localisation ORL du synovialosarcome est rare représentant moins de 5% des tumeurs de la région. Sa prise en charge est multidisciplinaire reposant sur une chirurgie large et complète suivie d'une radiothérapie externe. Nous rapportons deux cas de synovialosarcome de l'oropharynx et du sinus maxillaire chez deux adultes jeunes traités par une chirurgie et une radiothérapie externe avec une bonne réponse locorégionale.

## Introduction

Le synovialosarcome est une tumeur mésenchymateuse maligne rare, représentant 5% à 10% des sarcomes des tissus mous [1]. Il atteint principalement l'adolescent et l'adulte jeune, avec une atteinte préférentielle des membres [2]. La localisation au niveau de la sphère ORL est très rare, représentant moins de 5% des tumeurs de cette région [3], et siégeant essentiellement au niveau de l'hypopharynx et de l'espace parapharyngé. Le traitement standard repose sur l'exérèse large et complète, seule ou associée à une radiothérapie externe post opératoire [4]. Nous rapportons deux cas de synovialosarcome de l'oropharynx et du sinus maxillaire chez deux adultes jeunes traités par une chirurgie et une radiothérapie externe avec une bonne réponse locorégionale.

## Patient et observation

### Cas n^°^1

**Figure 1:**
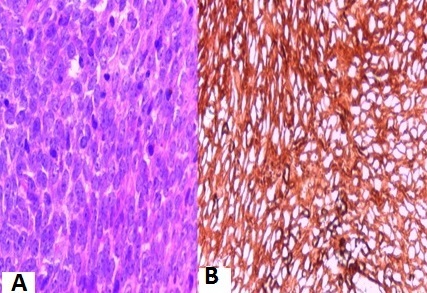
Images microscopiques objectivant, (A): une prolifération monophasique à cellules fusiformes, avec quelques figures mitotiques (HEx200); (B): un marquage positif par l'anticorps anti-CD99

Il s'agit d'une patiente âgée de 23 ans, sans antécédents pathologiques notables, qui a présenté une année avant sa consultation une otalgie gauche associée à une odynophagie. L'examen clinique et nasofibroscopique a objectivé une lésion bourgeonnante au niveau de la paroi postérieure de l'oropharynx sans adénomégalie cervicale associée. Le bilan d'extension comprenant une imagerie par résonance magnétique cervicale (IRM) a objectivé un processus lésionnel tissulaire hétérogène de 6 cm au niveau de la paroi postérieure de l'oropharynx, étendu en bas à l'hypopharynx, en isosignal T1 par rapport au muscle, en hyposignal T2, sans adénopathies cervicales associées. Une Tomodensitométrie thoracique dans le cadre du bilan d'extension à distance était normale. Une résection en monobloc de la tumeur sans curage ganglionnaire a été réalisée. L’étude anatomopathologique de la pièce opératoire à objectivé une prolifération mésenchymateuse fusicellulaire dont l'aspect était en faveur de sarcome. Le complément immunohistochimique a révélé un marquage positif des cellules tumorales à la cytokératine, EMA, vimentine et au PS 100, avec un absence de marquage par AML, CD34 et Desmine. Le diagnostic de synovialosarcome grade II de la FNCLCC a été retenu (Figure 1). Les limites de résection étaient positives. Une reprise chirurgicale proposée était difficile vu la localisation de la tumeur. La patiente a bénéficié d′une radiothérapie adjuvante à la dose de 70 Gy, en fractionnement de 2 Gy par jour, délivrée par deux champs latéraux, aux photons X de haute énergie de 6 MV. La patiente est actuellement en bon contrôle locorégional et à distance après 2 ans de la fin de traitement sans toxicité tardive notable.

### Cas n^°^2

**Figure 2:**
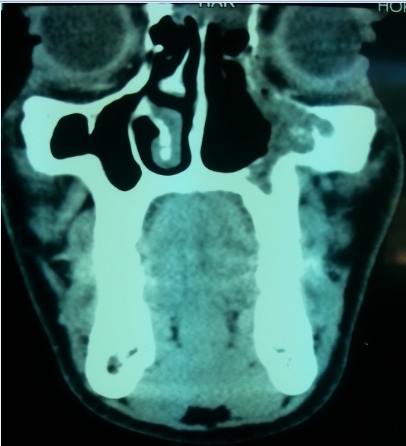
Coupe scanner en reconstruction coronale montrant une lésion tissulaire au niveau du sinus maxillaire gauche

**Figure 3:**
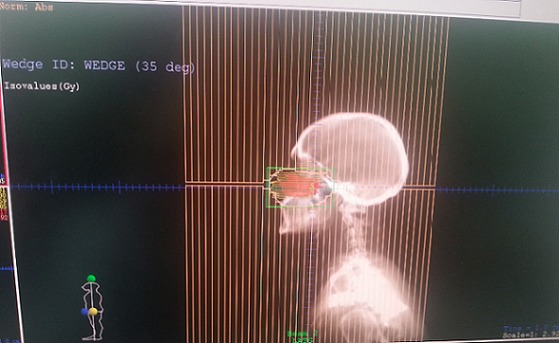
DRR (Digitally Reconstructed Radiographs) des champs d'irradiation utilisés: deux faisceaux latéraux (cas n 2)

Il s'agit d'un patient de 31 ans, sans antécédents pathologiques particuliers, qui a présenté une année avant sa consultation une tuméfaction au niveau du sinus maxillaire gauche qui augmentait progressivement de volume. Le bilan diagnostic comprenant une tomodensitométrie du massif facial a objectivé une masse tissulaire hétérogène de 6 cm au niveau du sinus maxillaire gauche sans adénopathie associée, le bilan d'extension était normal. (Figure 2) Le patient a bénéficié d'une chirurgie d'exérèse large mais fragmentée de la tumeur sans curage ganglionnaire prophylactique associé. L'aspect histologique de la lésion était en faveur d'une prolifération mésenchymateuse fusocellulaire. L’étude immunohistochimique a montré un marquage positif au vimentine, AE1 /AE3, PS100, CD99 et Bcl2, avec absence de marquage à l'EMA, desmine et la caldesmone, ce qui était en faveur d'un synovialosarcome, sans pouvoir préciser l’état des marges vu le caractère fragmenté de la pièce d'exérèse. Des recoupes qui ont été réalisées étaient normales. Une IRM post opératoire de contrôle n'a pas objectivé de résidu postopératoire. L'indication d'une radiothérapie adjuvante a été posée, sur la base de la taille de la tumeur et le caractère fragmenté de l'exérèse. La radiothérapie a été réalisée sur le lit tumoral, à une dose de 66 Gy, fractionnement de 2Gy par jour, en 2 champs latéraux droit et gauche aux photons de 6 Mv (Figure 3). Le patient est actuellement en bon contrôle loco régional et a distance après 10 mois de la fin de traitement.

## Discussion

Les sarcomes des tissus mous sont des tumeurs rares au niveau de la sphère ORL, représentant seulement 1% de toutes les tumeurs malignes de la tête et du cou [5]. Le synovialosarcome est une variété histologique de haut grade des sarcomes et il est considéré comme étant la quatrième entité la plus fréquente après les histiocytofibromes malins, les liposarcomes et les rhabdomyosarcomes [6]. Il intéresse surtout les adolescents et l'adulte jeune avec une nette prédominance masculine et un sexe ratio de 2,1 [7]. Sur le plan histologique, on distingue deux types de synovialosarcome: monophasique et biphasique. Dans le type monophasique, on retrouve uniquement des cellules fusiformes alors que dans le type biphasique on retrouve aussi bien des cellules épithéliales que fusiformes ce qui explique la différence par rapport aux autres sarcomes. Cette différence se reflète sur le plan immunohistochimique par la positivité des marqueurs épithéliaux: pancytokeratine (MAK-6), épithélial membrane antigen (EMA), et human epithelial antigen (Ber-EP4) [8]. Dans la dernière classification des tumeurs osseuses et des tissus mous de l'OMS, le synovialosarcome est classé parmi les tumeurs malignes à différentiation incertaine, pour equel un équivalent tissulaire sain est absent (WHO 2002). Sur le plan génétique, le synovialosarcome est caractérisé par la translocation chromosomique t(X;18) (p11;q11) qui en est spécifique. Cette anomalie est secondaire à la production par la tumeur d'une transcriptase polymérase reverse: SYT-SSX1; SYT-SSX2 [8]. Cette translocation n'a pas été recherchée chez nos deux patients. Malgré sa rareté, plusieurs facteurs pronostiques ont été suggérés qui sont principalement: l’âge, la localisation, la taille de la tumeur, un index mitotique élevé et un Ki-67 supérieur à 10% [9, 10]. Parmi ces derniers, chez nos patients, on retrouve la taille, l âge et la localisation ORL.

Sur le plan thérapeutique, le standard repose sur une exérèse large, complète en bloc de la tumeur. Bien que les marges chirurgicales soient bien définies dans les sarcomes des extrémités (qui sont de 5 cm); pour les sarcomes de la tête et du cou, vu la présence de structures vitales, il n'y a pas de standardisation des marges [4]. Néanmoins, la survie sans récidive y est fortement influencée. En effet la médiane de survie sans récidive est de 179 mois si elles sont négatives et de 33 mois si positives. (p < .0001) [11]. Malheureusement, chez la première patiente, une chirurgie complète n'a pas pu être réalisée vue la localisation et la taille de la tumeur. Compte au curage ganglionnaire prophylactique, de nos jours sa place dans les sarcomes n'est pas clairement établie. Nos deux patients n'y ont pas bénéficié. La radiothérapie post opératoire occupe une place importante dans le panel thérapeutique du synovialosarcome de la tête et du cou, surtout en cas de marges positives, limites marginales ou pour une tumeur supérieure à 5 cm [12], mais vu la rareté de la tumeur, il n y a pas une dose standard. Notre premier cas rapporté a reçu 70 Gy, le deuxième a reçu 66 Gy sur le lit tumoral sans irradiation ganglionnaire prophylactique pour les deux cas. La place de la chimiothérapie néoadjuvante à base d'ifosfamide n'est pas encore bien définie, mais certains auteurs la préconisent surtout quand la tumeur intéresse l'hypopharynx ou le larynx, vu que l exérèse complète en bloc est le plus souvent difficile [10, 11]. Nos deux patients n'ont pas reçu de chimiothérapie. Récemment, quelques auteurs ont proposé le rôle possible du récepteur de facteur de croissance épidermique (EGFR) et le récepteur du facteur de croissance épithélial humain 2 (HER-2 / neu) dans la cancérogenèse du synovialosarcome ce qui suggérerait que la thérapie anti-EGFR peut jouer un rôle dans l'approche thérapeutique [8].

## Conclusion

Le synovialosarcome est une tumeur rare de la sphère ORL. Sa prise en charge doit être multidisciplinaire basée sur une chirurgie large, complète et une radiothérapie externe adjuvante afin d'améliorer le pronostic qui reste a l'heure actuelle mauvais vu le caractère agressif de la tumeur.
